# Assessing the Responses of Large Language Models (ChatGPT-4, Gemini, and Microsoft Copilot) to Frequently Asked Questions in Breast Imaging: A Study on Readability and Accuracy

**DOI:** 10.7759/cureus.59960

**Published:** 2024-05-09

**Authors:** Murat Tepe, Emre Emekli

**Affiliations:** 1 Radiology, Mediclinic City Hospital, Dubai, ARE; 2 Radiology, Eskişehir Osmangazi University Health Practice and Research Hospital, Eskişehir, TUR

**Keywords:** artificial intelligence, breast imaging, microsoft copilot, gemini, chatgpt, large language models

## Abstract

Background

Large language models (LLMs), such as ChatGPT-4, Gemini, and Microsoft Copilot, have been instrumental in various domains, including healthcare, where they enhance health literacy and aid in patient decision-making. Given the complexities involved in breast imaging procedures, accurate and comprehensible information is vital for patient engagement and compliance. This study aims to evaluate the readability and accuracy of the information provided by three prominent LLMs, ChatGPT-4, Gemini, and Microsoft Copilot, in response to frequently asked questions in breast imaging, assessing their potential to improve patient understanding and facilitate healthcare communication.

Methodology

We collected the most common questions on breast imaging from clinical practice and posed them to LLMs. We then evaluated the responses in terms of readability and accuracy. Responses from LLMs were analyzed for readability using the Flesch Reading Ease and Flesch-Kincaid Grade Level tests and for accuracy through a radiologist-developed Likert-type scale.

Results

The study found significant variations among LLMs. Gemini and Microsoft Copilot scored higher on readability scales (p < 0.001), indicating their responses were easier to understand. In contrast, ChatGPT-4 demonstrated greater accuracy in its responses (p < 0.001).

Conclusions

While LLMs such as ChatGPT-4 show promise in providing accurate responses, readability issues may limit their utility in patient education. Conversely, Gemini and Microsoft Copilot, despite being less accurate, are more accessible to a broader patient audience. Ongoing adjustments and evaluations of these models are essential to ensure they meet the diverse needs of patients, emphasizing the need for continuous improvement and oversight in the deployment of artificial intelligence technologies in healthcare.

## Introduction

Large language models (LLMs), such as ChatGPT-4, Gemini, and Microsoft Copilot, have revolutionized the field of artificial intelligence (AI) by demonstrating an unprecedented ability to understand and generate human-like text [1-3]. These chatbot models are trained on diverse internet datasets, allowing them to acquire vast amounts of knowledge and language nuances [4,5]. LLMs perform a variety of tasks, from answering queries to generating coherent and contextually appropriate responses, making them potent tools for information dissemination and decision support across multiple domains [6,7].

Breast imaging is a crucial component of diagnostic medicine, aiding in the early detection and management of breast diseases, notably cancer. Techniques such as mammography, ultrasound, and MRI are routinely used to screen and diagnose millions of patients worldwide. However, the increasing demand for these diagnostic services places a significant strain on healthcare systems, often leading to overwhelming workloads for radiologists and associated healthcare workers [8]. This surge underscores the need for efficient, scalable solutions to manage patient queries and enhance service delivery.

Health literacy is fundamental to empowering patients, enabling them to make informed decisions regarding their healthcare. In the context of breast imaging, understanding the purposes, processes, and potential outcomes is vital for patients as it directly influences their engagement and compliance with screening programs [9]. High levels of health literacy contribute to better patient outcomes, reduced anxiety, and more efficient use of healthcare resources, yet many individuals struggle to find reliable, understandable information [10].

LLMs have the potential to significantly improve the patient experience by providing instant, reliable, and easily understandable answers to common questions regarding breast imaging. By leveraging their vast training data, these models can offer explanations, guidelines, and reassurance about procedures, thus enhancing health literacy [11,12]. This capability not only aids patients in navigating their health choices but also alleviates some of the informational burdens shouldered by medical staff.

As AI technology continues to permeate the healthcare sector, understanding its capabilities and limitations is crucial. This research will provide insights into the feasibility of using LLMs to enhance patient understanding of complex breast imaging procedures, ultimately contributing to more informed patient choices and better health outcomes. The objective of this research is to evaluate the readability and accuracy of the information provided by LLMs in response to frequently asked questions by patients about breast radiological imaging.

## Materials and methods

When selecting the sample questions for our study, we compiled the 20 most frequently asked questions by patients in real life. To select the most relevant and frequently asked questions for our study on breast imaging, we employed a two-step process involving both technological and expert assessments. Initially, we utilized Google Trends to identify common queries related to breast imaging, leveraging this tool to reflect current public interest and common concerns. Subsequently, we compiled an initial list of 35 questions based on the data from Google Trends combined with the clinical experiences of two radiologists, each with four to seven years of experience in breast radiology, to ensure questions were medically pertinent. To refine this list, an expert panel consisting of four radiology specialists with seven, four, two, and two years of experience in breast radiology was formed. The panel employed a structured voting process to evaluate the questions. Each expert independently rated the relevance and frequency of each question. Questions were then discussed collectively, and a consensus was required for a question to be included in the final set. Finally, the top 20 questions most likely to be encountered in clinical practice were selected (Table 1).

**Table 1:** Frequently asked questions by patients regarding breast imaging. Q = questions

Questions	
Q-1	At what age should I begin breast cancer screening?
Q-2	Several members of my family have previously been diagnosed with breast cancer. For this reason, should I undergo breast screening more frequently?
Q-3	Is there a risk of radiation exposure from having regular mammograms?
Q-4	Does breast cyst go away on its own?
Q-5	How to detect the presence of breast cancer?
Q-6	Can I get a mammogram that doesn’t compress my breast?
Q-7	Would you recommend a breast MRI or ultrasound over a mammogram?
Q-8	My mammogram report said that I have dense breast tissue. What does this mean?
Q-9	Will getting a mammogram damage my breast implants?
Q-10	How to understand whether a breast mass is dangerous with imaging?
Q-11	Does the mammogram definitively show whether the breast mass is good or bad?
Q-12	Do all breast masses need to be biopsied?
Q-13	Can it be understood that the breast mass is good or bad without taking a biopsy?
Q-14	Can a breast MRI be used instead of a biopsy?
Q-15	If I get a breast ultrasound or an MRI, can I stop doing yearly mammograms?
Q-16	What are the risks of a breast biopsy?
Q-17	Is a breast biopsy a surgery-like procedure?
Q-18	If the breast mass is malignant, will the cancerous cells spread while the biopsy is taken?
Q-19	Does the breast biopsy give definitive results?
Q-20	Can a benign breast lesion become malignant in the future?

The questions were submitted once each to ChatGPT-4, Gemini, and Microsoft Copilot on April 12, 2024, and the responses were recorded. No other specific prompts were used to enhance the responses of the chatbots. For every new search request made in chatbots, a separate conversation page was initiated to prevent past queries from influencing the responses to subsequent queries. As the design of the study did not involve any real patient data, ethical committee approval was not sought.

Readability assessment of the chatbot responses

To quantitatively evaluate the readability of responses of LLMs, we used two readability tests designed to indicate how difficult a passage in English is to understand. For this analysis, we calculated and recorded the Flesch Reading Ease (FRE) and Flesch-Kincaid Grade Level (FKG) readability scores for each response obtained from LLMs to the frequently asked questions about breast imaging. The FRE score is determined based on the aggregate number of words, sentences, and syllables within the text, calculated using the following formula: FRE = 206.835 - (1.015 × (total words/total sentences)) - (84.6 × (total syllables/total words)) [13]. According to this index, texts with shorter sentences and fewer syllables per word are deemed more readable. Scores on the FRE scale range from 90-100 for very easy, 80-89 for easy, 70-79 for fairly easy, 60-69 for standard, 50-59 for fairly difficult, 30-49 for difficult, to 0-29 for very confusing texts. The FKG formula calculates the grade level necessary for understanding the text, with the initial step involving computing the average sentence length (ASL) and the average number of syllables per word (ASW). The resulting formula is FKG = (0.39 × ASL) + (11.8 × ASW) - 15.59 [14]. The score derived from this calculation corresponds to the educational grade level, as categorized in the US educational system. For example, a score of 8.0 means that the text is expected to be understandable by an average eighth grader. Texts with lower scores are easier to read, while texts with higher scores are more complex.

Accuracy and appropriateness of the chatbot responses

To evaluate the accuracy and appropriateness of the responses received from LLMs, we created a Likert-type scale ranging from 1 to 5 (Table 2). This scale was developed through a consensus between two radiologists with four and seven years of experience in breast imaging. In the development of this Likert scale, multiple critical dimensions were taken into account: (1) scientific accuracy, which evaluates whether the information aligns with current scientific knowledge; (2) relevance, assessing whether the information directly addresses the patients’ questions; and (3) actionability, determining whether the information includes clear, practical guidance or steps that patients can implement based on the provided data. Using the developed Likert scale, each response provided by the chatbots was scored based on the consensus formed by two radiologists.

**Table 2:** Likert scale to assess the accuracy and appropriateness of chatbot responses.

Score	Accuracy	Description
1	Completely inaccurate	The material contains numerous factual errors, misleading information, or misconceptions that could potentially harm the patient’s understanding or health outcomes
2	Somewhat inaccurate	While there are some correct elements, the material has significant inaccuracies or omissions that might confuse patients or lead to misunderstandings about their health outcomes
3	Moderately accurate but lacks clarity or depth	The information is generally accurate but it lacks sufficient detail on critical points, which could hinder effective self-care or decision-making
4	Mostly accurate	The material provides accurate information in a clear and understandable manner but may have minor inaccuracies or areas where additional clarification could enhance the patient’s health outcomes
5	Highly accurate	The information is accurate and well-researched. It comprehensively addresses the topic, enabling patients to fully understand the issue without misconceptions or significant questions remaining

Statistics

Statistical analyses were performed using SPSS for Windows, version 25.0 (IBM Corp., Armonk, NY, USA). Descriptive statistics are expressed as the mean and standard deviation for accuracy scores and readability scores. The Shapiro-Wilk test, kurtosis, and skewness values were used to assess normality. A normal distribution was accepted if kurtosis and skewness values were between (-1.5) and (+1.5). Levene’s test was used to examine variance homogeneity. One-way analysis of variance was applied to determine the interactions between chatbots for accuracy scores and readability scores, and post-hoc tests were performed to make pairwise comparisons between each chatbot.

## Results

Readability scores

**Figure 1:**
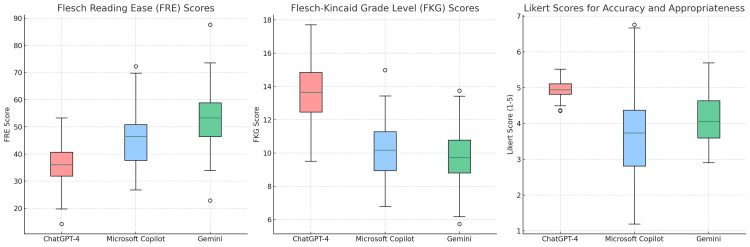
The scores of LLMs in terms of readability and accuracy are shown on a boxplot. Higher FRE and lower FKG scores indicate easier readability. LLM = large language model; FRE = Flesch Reading Ease; FKG = Flesch-Kincaid Grade Level

The FRE readability scores for ChatGPT-4, Microsoft Copilot, and Gemini were 37.15 ± 8.74, 45.6 ± 9.81, and 52.45 ± 9.12, respectively, while the FKG scores were 13.55 ± 1.9, 10.3 ± 1.52, and 9.92 ± 1.69, respectively. Significant differences were observed among the chatbots in terms of both FRE and FKG scores (p < 0.001 and p < 0.001, respectively). Specifically, ChatGPT-4 exhibited statistically lower FRE scores compared to Microsoft Copilot and Gemini (p = 0.015 and p < 0.001, respectively) and statistically higher FKG scores (p < 0.001 and p < 0.001, respectively). However, there were no significant differences between Microsoft Copilot and Gemini in terms of both FRE and FKG scores (p = 0.058 and p = 0.761, respectively) (Figure 1) (Appendix A).

Accuracy and appropriateness of the chatbot responses

The Likert scores were calculated as 4.95 ± 0.22 for ChatGPT-4, 3.65 ± 1.18 for Microsoft Copilot, and 3.95 ± 0.69 for Gemini. There was a statistically significant difference among the scores for the three chatbots (p < 0.001). When compared to Microsoft Copilot and Gemini, the score of ChatGPT-4 was statistically higher (p < 0.001 and p < 0.001, respectively). However, there was no significant difference between Microsoft Copilot and Gemini (p = 0.594) (Figure 1) (Appendix B).

## Discussion

The findings from this study underscore the potential for LLMs, such as ChatGPT-4, Microsoft Copilot, and Gemini, to enhance health literacy in the field of breast imaging. The results demonstrate significant variations among these models in terms of readability and the accuracy of the responses to common patient inquiries about breast imaging. This variation highlights the importance of selecting the right tool for disseminating complex medical information in a manner that is both accessible and reliable.

The readability assessments revealed that Gemini and Microsoft Copilot exhibited the highest FRE and lowest FKG scores, indicating that they produced the most easily comprehensible responses compared to ChatGPT-4. This finding is crucial because it suggests that the responses from Gemini and Microsoft Copilot are not only easy to read but also, considering educational levels, potentially address a broader patient population compared to the responses from ChatGPT-4. In the literature, there are conflicting results regarding this subject. Hillmann et al. [15] posed questions related to atrial fibrillation and cardiac implantable electronic devices to various chatbots, and similarly to our study, found that ChatGPT scored lower in terms of readability. However, Mu et al. [16] and Seth et al. [17] asked questions related to melanoma and rhinoplasty, respectively, to chatbots, and while the first study found no significant difference in readability among the chatbots, the other study conducted by Seth et al. found ChatGPT and BARD (now called Gemini) to be superior in terms of readability. Haver et al. [18], using the ChatGPT-3.5 version, requested to simplify the answers given to questions about breast cancer prevention and screening by entering an additional prompt into ChatGPT, and found that ChatGPT’s responses were statistically significantly simplified compared to the original ones. However, in our study, the original responses received without entering such an extra prompt were evaluated. Given the continuous and rapid changes and improvements in LLM technology, it is clear that more comprehensive research will be needed in the future.

In terms of accuracy of responses, while all three chatbots demonstrated commendable performance, ChatGPT-4 significantly outperformed both Microsoft Copilot and Gemini. In several studies where only ChatGPT was tested, it has generally been found to be successful in answering frequently asked questions by patients in various medical fields [18-21]. These results are also consistent with our study. Furthermore, there is a need for more studies that test and compare the responses of LLMs in the field of medical communication in terms of accuracy and appropriateness.

LLMs, including the ones assessed in our study, are trained on extensive and diverse corpora that inherently contain biases present in the original source material. These biases can manifest in skewed responses, especially in specialized fields such as breast imaging. Another drawback of LLMs is that they can generate responses that appear reliable but are inaccurate. This issue is often referred to as the “hallucination effect” [22]. Additionally, LLMs can generate different answers to the same question upon repeated queries [23]. However, in our study, the decision to query each question only once was primarily driven by the need to maintain consistency and manageability within the experimental design. Future research could, therefore, benefit from multiple iterations of the same queries to assess the consistency of LLM outputs.

These findings suggest several implications for the deployment of LLMs in responding to patient inquiries about breast imaging. First, there is a clear need for ongoing evaluation and calibration of these models to ensure they meet the specific needs of different patient populations with a variety of educational backgrounds. Second, the reliance on LLMs also necessitates rigorous oversight to maintain the quality of information and to update it in line with evolving medical standards and practices. Lastly, the study reflects the broader impact of AI in healthcare, potentially enhancing patient engagement and health literacy. By improving understanding, LLMs can help bridge the gap in health communication, particularly in areas such as breast imaging where patient awareness and understanding are critical to early detection and treatment success.

This study has several limitations. First, the search was restricted to 20 questions. The formulation of inputs when interacting with LLMs can significantly affect the quality and nature of the generated responses. Moreover, it remains a subject of debate whether LLMs consistently produce identical or similar responses to the same query at different times. In this study, each question was submitted only once to the chatbots. Furthermore, while the readability of the responses was assessed, the absence of real patients as evaluators in this aspect constitutes a limitation of the study.

## Conclusions

While ChatGPT-4 can produce more accurate answers to frequently asked questions about breast imaging, its readability scores remain lower compared to Microsoft Copilot and Gemini. Considering their continuous and rapid development, it is inevitable that in the future, chatbot responses in the medical field will become even more accurate and that chatbot systems capable of providing responses tailored to the literacy levels of the readers will be developed. As a result, the use of LLMs in medicine is bound to become more frequent. Further research should explore the longitudinal effects of LLM interaction with patients and its impact on health outcomes, as well as information dissemination.

## Appendices

Appendix A. Readability scores of the chatbot responses

**Table 3:** Readability scores of the chatbot responses.

	Readability-Flesch Reading Ease			Readability-Flesch-Kincaid Grade Level		
	ChatGPT-4	Gemini	Copilot	ChatGPT-4	Gemini	Copilot
1	48	55	58	12.71	9.78	10.04
2	39	50	56	13.10	10.55	8.72
3	29	43	45	15.97	11.66	10.46
4	45	63	65	13.13	8.58	6.37
5	50	61	50	10.11	7.68	9.19
6	35	61	53	14.36	8.48	8.8
7	46	56	41	10.73	8.95	10.15
8	46	53	54	13.04	10.07	8.67
9	40	59	45	12.45	8.43	11.53
10	30	36	30	12.94	12.44	10.76
11	40	39	31	12.42	12.04	11.9
12	33	59	61	14.25	7.74	8.84
13	35	38	36	14.06	13.20	12.09
14	41	58	46	13.11	8.99	10.12
15	41	54	44	13.57	11.33	10.31
16	45	67	40	12.12	7.50	11.98
17	29	40	42	14.08	11.25	11.63
18	30	55	42	14.59	9.28	11.92
19	24	46	34	15.19	11.1	10.49
20	17	56	39	19.11	9.31	12.04

Appendix B. Accuracy scores of chatbot responses according to the Likert-type scoring system

**Table 4:** Accuracy scores of chatbot responses according to the Likert-type scoring system.

Likert scale scores			
	ChatGPT-4	Gemini	Copilot
1	5	4	4
2	5	4	3
3	5	4	5
4	4	4	4
5	5	4	4
6	5	5	3
7	5	3	5
8	5	5	5
9	5	3	5
10	5	4	2
11	5	4	3
12	5	4	3
13	5	5	4
14	5	4	3
15	5	3	5
16	5	4	4
17	5	3	3
18	5	4	5
19	5	3	2
20	5	5	1
